# Thermodynamics of the Densification Process for Polymer Glasses

**DOI:** 10.6028/jres.081A.018

**Published:** 1977-04-01

**Authors:** John E. McKinney, Robert Simha

**Affiliations:** Institute for Materials Research, National Bureau of Standards, Washington, D. C. 20234; Department of Macromolecular Science, Case Western Reserve University, Cleveland, Ohio 44106 (May 3, 1977)

**Keywords:** Compressibility, densification, glass, glass transition, liquid, polymer, pressure, *PVT*, pyrolysis, refractive index, thermal expansion, thermodynamic

## Abstract

A quantitative description is given for the densification process of glasses resulting from glass formation at elevated pressures. Phenomenologieal relations are derived, or justified, which allow estimation of the densification rate *κ′* (with respect to formation pressure) from various thermodynamic quantities and glass transition behavior. In addition, the estimation of *K′* may be facilitated by the application of the hole theory of Simha and Somcynsky. Using these relations *κ′* is estimated, and the results from the different methods are compared for data from 23 different organic polymers with glass transition temperatures ranging from 150 to 455 K. The amount of densification appears to be limited by the apparent convergence of the glass temperature and effective decomposition temperature with increasing pressure. Some estimates of limiting values are presented. Finally, changes of refractive index resulting from densification are estimated from the observed, or predicted, densification rates.

## 1. Introduction

**Figure 1:**
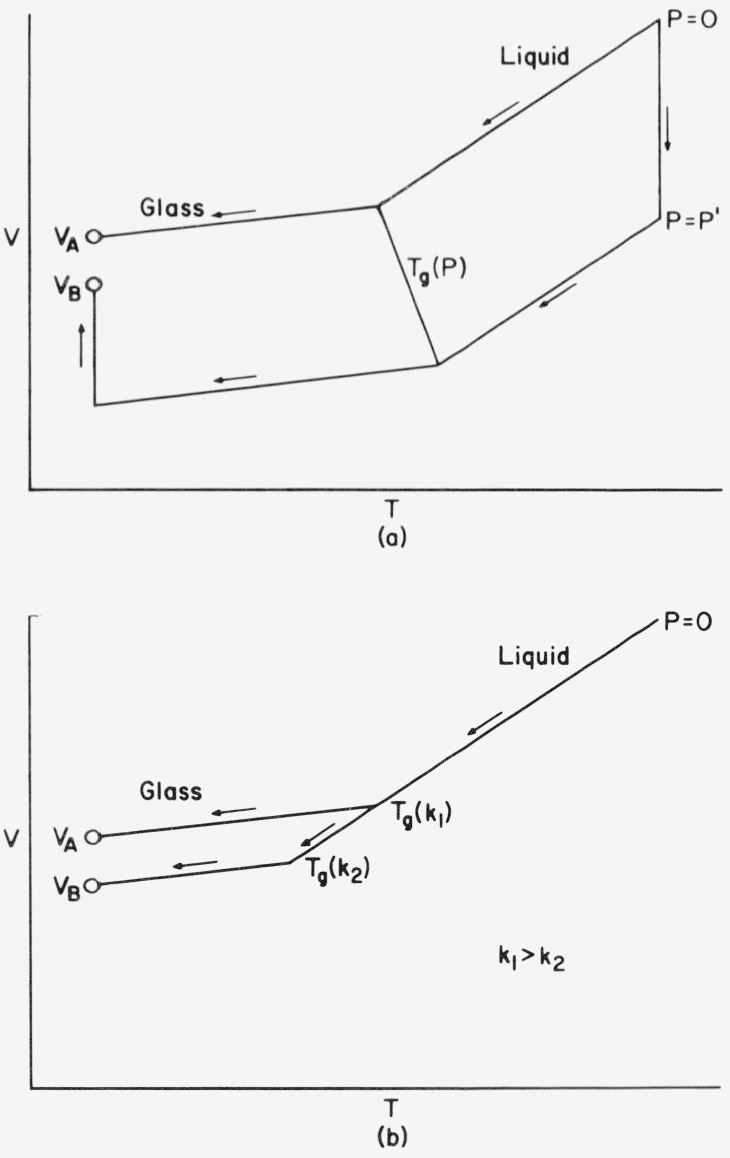
Schematic illustration of two methods used to obtain densijied glasses. (a) Densification by elevating the formation pressure at the same cooling rate k_1_. (h) The same densification is obtained by commensurately decreasing the cooling rate at atmospheric pressure.

The density of a glass, as well as certain other properties, depend upon the thermodynamic history by which the glass is formed. For example, as shown schematically in figure 1a, an amorphous polymer subjected to an elevated pressure in the melt, followed by isobaric cooling at constant rate to a temperature well below the glass temperature, *T_g_*, and then depressurized, will have a larger density than that obtained by isobaric cooling at the same rate at atmospheric pressure to the same temperature in the glass. From the former procedure the pressure induced densification rate is defined as

$$\kappa^{\prime }=-\left(1/V\right){\left(\partial V/\partial P^{\prime }\right)}_{T,P,k}$$ (1)

where *V* is the volume at temperature *T* and pressure *P*, and *P'* is the formation pressure maintained during constant rate of cooling *k*. Note that this definition parallels the usual one for the isothermal compressibility,

$$\kappa =-\left(1/V\right){\left(\partial V/\partial P\right)}_{T,P^{\prime },k,}$$ (2)

the difference being that *P* and *P′* are interchanged.

It is expected that the final depressurized volume in the glass will lie between the atmospheric and the pressurized value, as shown in figure 1a. It is then clear that the inequality,

$$\kappa_{g}>\kappa^{\prime }>0$$

where *κ_g_* is the compressibility of the glass, is obeyed. Although we may intuitively expect this relation to hold, as apparent from experiment, we do not know of any proof.

A well known alternative method of densifying glasses is simply to decrease the cooling rate as illustrated in figure 1b. In principle one can obtain the same volume in the glass by this procedure as by elevating the pressure, except that the times required for the former are much longer. For example, it is estimated [1]^1^ that a poly (vinyl acetate) glass obtained by isobaric cooling at 800 bar^2^ in 8 hours would require 500 years to reach the same volume at the same terminal temperature by cooling slowly at atmospheric pressure. It should be recognized, however, that the states of glasses at, the same volume, temperature, and pressure, but obtained through different histories, are not necessarily the same. As pointed out by Bree and coworkers [2], volume changes during isobaric-isothermal volume relaxation [3] have a large effect on relaxation times for creep compliance, whereas almost no effect is observed from volume changes obtained by isobaric cooling at elevated pressures. Accordingly, it appears that the state of a glass is not determined by its volume, temperature, and pressure alone. Moreover, pressure induced densification does have an influence on physical properties. According to the data of Dale and Rogers [4] over a 5 kbar range, the compressive modulus of polystyrene appears to increase slightly with formation (or molding) pressure, leveling off at higher pressures, with the yield stress going through a maximum between 1 and 1.5 kbar. Wetton and Money-penny [5] have studied the dynamic mechanical and dielectric properties of several polymeric glasses formed at pressures up to slightly beyond 5 kbar. Both the real part of Young’s modulus and its loss tangent, as well as the real part of the dielectric constant, increase with formation pressure. For polyvinyl acetate) McKinney and Goldstein [1] have observed a 3 percent increase in the bulk modulus at 0 ° C, corresponding to a formation pressure of 800 bar. This difference increases with decreasing temperature.

Thermal properties also seem to depend on the amount of pressure induced densification. Although the heat capacity *C_p_* is found to be independent [6] of formation pressure, the enthalpy *H* seems to vary significantly at formation pressures above a certain value. According to Price [7], very little change in the enthalpy of poly(methyl methacrylate) is observed up to about 800 bar, followed by a nearly constant rate of increase of about 0.015 cm^3^/g up to 3 kbar, their maximum value. For polystyrene [8], the data have been evaluated as Δ*H = H*(densified) − *H*/(normal) first decreasing slightly and then going back to zero at about 800 bar, followed by an increase with nearly constant slope up to the maximum pressure. Weitz and Wunderlich [9] have also observed this behavior and interpreted it in terms of two opposing mechanisms arising from holes and rotational isomers. It is not clear, however, that the apparent negative values of Δ *H* obtained by experiment are significant.

The purpose of this paper is to describe the thermodynamics of the pressure induced densification process by applying both phenomenological and molecular theory. Simple phenomenological relationships are derived between the densification rate *κ′* and other thermodynamic properties for which values are more readily available in the literature. Moreover, it is shown how the hole theory of Simha and Somcynsky [10] may be used to facilitate the estimation of the densification rates for polymers using a minimum amount of experimental information. In both cases the derived relationships are tested using appropriate experimental data. An example of the utility of these results is demonstrated by estimating the change in the index of refraction corresponding to changes in molding pressure, assuming that the index of refraction is related to the volume by the Lorentz-Lorenz equation. The results have potential application to the adjustment of the refractive indices of lenses by varying the molding pressure.

## 2. Phenomenological Relationships

**Figure 2:**
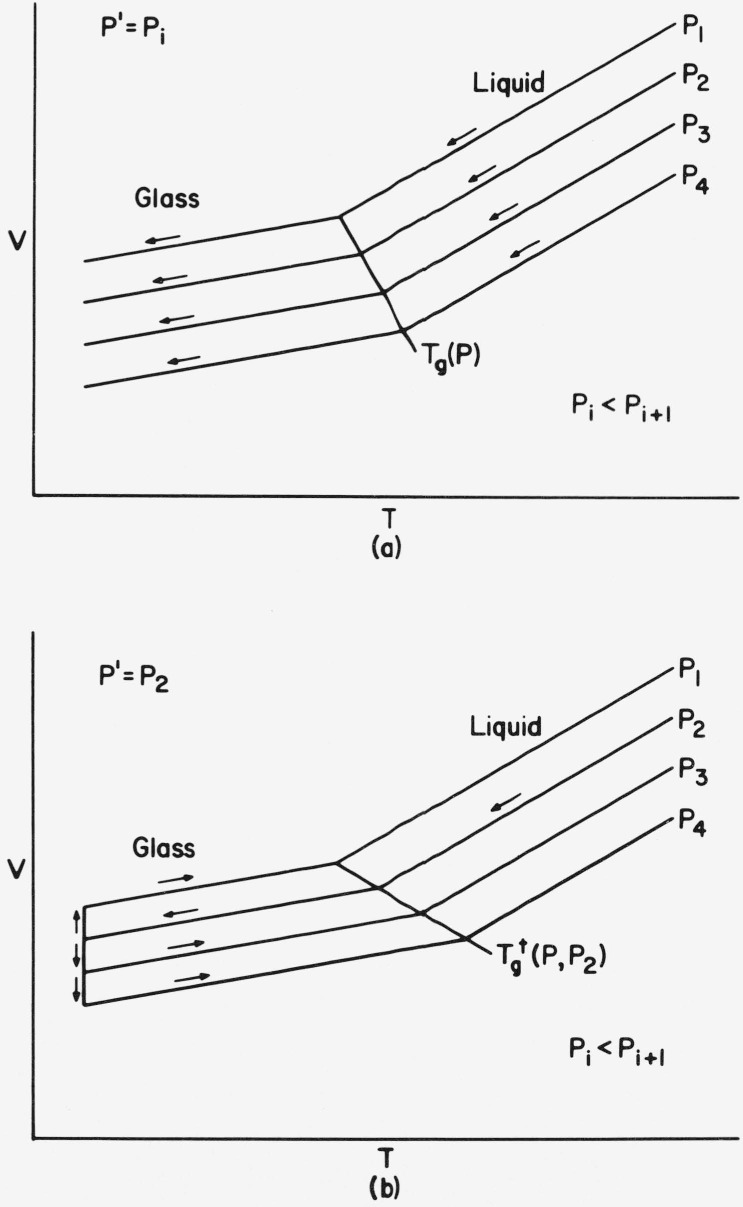
Schematic illustration of two thermodynamic histories used to form glasses. (a) Variable formation, (b) Constant formation.

Two types of thermodynamic histories, shown schematically in figure 2, are pertinent to the development of the phenomenological relationships for pressure induced densification. In the first (Fig. 2a) the *PVT* surface of the glass is obtained from repeated isobaric cooling runs at the same constant rate, but at difference pressures, with all pressure changes occurring in the melt prior to each run. This procedure is called the variable formation history because the structure of the glass is different for each experimental pressure (which is the formation pressure, since *P* = *P′)*. The glass transition at each pressure is assumed to occur at a constant mean relaxation time. Hence, the intersection of the liquid and glass *PVT* surfaces gives the proper *T_g_*(P), from which *dT_g_/dP* is expected to approximate that obtained from the dynamic mechanical and dielectric frequency-temperature-pressure superposition. On the other hand, as a consequence of the varied structure, the glass *PVT* surface is not proper in the thermodynamic sense.

With the other history (fig. 2h) the glass is formed also by isobaric cooling at constant rate at an arbitrary pressure, *P′* (which is usually atmospheric, but elevated in figure 2b to illustrate the more general case). At temperatures well below *T_g_*, where viscoelastic relaxation times are large in comparison to effective experimental times, a thermodynamically reversible *PVT* surface for the glass is obtained by observing the volumetric response to “fast” changes in temperature and pressure. Since all of the data in the glass pertain to the same *P′*, the *PVT* surface gives the proper values of the derivable thermodynamic quantities (for example, thermal expansion, isothermal compressibility, and internal pressure). The intersection of the liquid and glass surfaces defines the fictive temperature $T_{g}^{†}\left(P,P^{\prime }\right)$.

The principal distinction in procedure between the two histories is that with variable formation all pressure changes are made in the melt, whereas with constant formation they are made in the glass. Note that the number of independent variables is different for *T_g_(P)* and $T_{g}^{†}\left(P,P^{\prime }\right)$. The redundancy of using two arguments in the former arises from the fact that the formation and experimental pressure are always identical. Accordingly, *T_g_* may be regarded as a special case of $T_{g}^{†}$ when *P = P′*. The implicit argument *k* is deleted here because only one value appplies to these discussions for each case. For further details and interpretations of these histories, see Ref. 11 ].

In all of the schematic diagrams in this paper the glass transition is shown as a discrete intersection. With isobaric cooling at constant rate through *T _g_* a gradual transition process is observed. The discrete intersections shown correspond to those obtained by extrapolation of the equilibrium isobars and the isochronal (nonrelaxing) ones for the glass.

**Figure 3:**
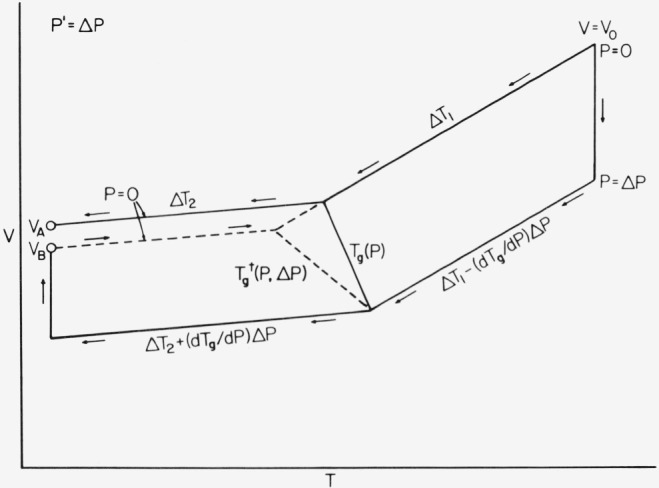
Schematic illustration of the procedure used to derive the densification equation [See eqs (3) and (4)], and the distinction between T_9_(P) and $T_{g}^{†}\left(P,P^{\prime }\right)$.

We now proceed to evaluate the thermodynamic diagram in figure 3, in order to determine relationships for *κ′* in terms of other measured quantities. Volume *A* is obtained by isobaric cooling at constant rate and atmospheric pressure *(P = P′* = 0). Volume *B* is reached by pressurizing to *P = P′* = Δ *P* in the melt, followed by isobaric cooling at the same constant rate as for *A*, with subsequent depressurizing in the glass at the same terminal temperature as for *A*. Note that *T_g_* (in lieu of $T_{g}^{†}$) applies here, since the transition is observed at the formation pressure (*P* = *P*′) in both cases. The isobaric extension of *V_B_* with increasing *T* (see dashed line) to its intersection with the liquid line yields the fictive temperature $T_{g}^{†}\left(P,P^{\prime }\right)=T_{g}^{†}\left(O,\Delta P\right)$.

In summing the thermodynamic contributions for small changes in *T* and *P* near *T_g_(P)* in the range where linear approximations are valid, we find

$$\begin{array}{l} V_{A}=V_{0}\left(1-\alpha_{l}\Delta T_{1}-\alpha_{g}\Delta T_{2}\right)\\ V_{B}=V_{0}\left\{1-\kappa_{l}\Delta P-\alpha_{l}\left[\Delta T_{1}-\left(dT_{g}/dP\right)\Delta P\right]-\alpha_{g}\left[\Delta T_{2}+\left(dT_{g}/dP\right)\Delta P\right]+\kappa_{g}\Delta P\right\} \end{array}$$ (3)

where *α* is the usual isobaric thermal expansivity, the Δ’s indicate differences as shown on figure 3, and the subscripts *l* and *g* pertain to liquid and glass. For small changes eq (1) may be written in the form

$$\kappa^{\prime }=\left(V_{A}-V_{B}\right)/\left(V_{0}\Delta P\right)$$ (4)

Substitution of eqs (3) for *V_A_* and *V_B_* yields

$$\kappa^{\prime }=\Delta \kappa -\Delta \alpha dT_{g}/dP$$ (5a)

where the Δ ’s here indicate the usual differences in the respective quantities between liquid and glass. Since

$$dT_{g}^{†}/dP=\Delta_{k}/\Delta \alpha$$ (6a)

along $T_{g}^{†}$, [11], *κ′* may be expressed in terms of the difference between the two transition rates, i.e.,

$$\kappa^{\prime }=\Delta \alpha \left(dT_{g}^{†}/dP-dT_{g}/dP\right)$$ (5b)

Equation (5a) may also be written as an Ehrenfest-type relation, viz.,

$$dT_{g}/dP=\Delta \kappa /\Delta \alpha -\kappa^{\prime }/\Delta \alpha$$ (5c)

which is consistent with the experimentally observed inequality

$$dT_{g}/dP\leq \Delta \kappa /\Delta \alpha$$

provided the densification rate is non-negative. Expressions fully equivalent to eqs (5) have been derived by Goldstein [11] and given previously in Ref. [12]. From eqs (5) it is evident (as also pointed out by Goldstein [11]) that the necessary and sufficient condition (assuming Δα ≠ 0) for the *PVT* surface to be independent of formation pressure is the validity of the first Ehrenfest equation

$$dT_{g}/dP=\Delta \kappa /\Delta \alpha .$$ (6b)

The analogous argument applies to the entropy surfaces. Since the second Ehrenfest equation,

$$dT_{g}/dP=T_{g}V\Delta \alpha /\Delta C_{P}$$ (7)

where *C_P_* is the usual heat capacity at constant pressure, appears to be a good approximation [11, 13], there should be a single entropy surface with respect to formation pressure in contrast to the manifold/surface observed for volume. This view is confirmed by the DSC^3^ measurements of Yourtee and Cooper [6] on normal and densified polystyrene, which reveal no significant effect on the thermal properties of glasses by vitrification at elevated pressures. The authors did find some differences in the thermal behavior between these properties and those from vitrification by isothermal compression; however, these were attributed to inhomogeneous freezing processes during compression. Accordingly, if eq (7) is a good approximation, it leads to a convenient experimental determination of the initial *(P* = 0) value of *dT_g_/dP* through volume-temperature and heat capacity measurements required at atmospheric pressure only. Equation (7) will be tested by means of experimental data later in this paper.

As stated above *κ′* may be determined (near *T_g_*) from the values of Δ *α*, *dT_g_/dP*, and Δ*κ* using eq (5a). The relative difficulty in obtaining these quantities experimentally increases in the order given above, as does the difficulty of obtaining their values from the literature. For these reasons it is desirable to be able to estimate Δ*κ* (or Δ *κ*/Δ *α*) independently of existing PVT data. It will be shown how the hole theory of Simha-Somcynsky [10] may be used to arrive at values of $dT_{g}^{†}/dP=\Delta \kappa /\Delta \alpha$.

As indicated previously, eqs (5) are based on several linearizations. It is assumed that the coefficients *α_l_, α_g_, κ_l_* and *κ_g_* are independent of pressure and temperature and that *T_g_* is a linear function of pressure. Thus strictly, the reference temperature in the glass as well as the initial temperature in the melt should be appropriately close to *T_g_*. Moreover, the pressure *P′* should be appropriately small. In the Appendix the general relationships are developed, based on the equations of state of the liquid and both glasses.

As an example, integral relations are evaluated over the two paths shown on figure 1a for PVAc, for which extensive data are available [1], and the Tait parameters are known [14] for the liquid and both glasses. The results are tabulated and compared with the corresponding linear approximations.

## 3. Application of Molecular Theory

The hole theory, which is used here to estimate the values of $dT_{g}^{†}/dP$, is a corresponding states theory based on a lattice model. The partition function is defined in terms of a single ordering parameter, the hole fraction *h*, which gives the ratio of the number of vacant to total sites, each of which may be occupied by a polymer segment. The corresponding states are given in terms of the reduced (universal) variables

$$\tilde{T}=T/T^{*},\tilde{P}=P/P^{*},\tilde{V}=V/V^{*}$$ (8)

where T*, P*, and V* are the scaling factors applicable to each polymer. Although these are defined explicitly by the theory, they are usually derived from a superposition of equilibrium *PVT* data along the master curves evaluated from the theory. For an illustration of this procedure, see Ref. [14].

The partition function *Z* is expressed uniquely in terms of the three independent variables $\tilde{T}$, $\tilde{V}$ and *h*. From the thermodynamic definition

$$P=-kT{\left[\partial \ell nZ\left(T,V,h\right)/\partial V\right]}_{T}$$

and the equilibrium constraint *(∂Z/∂h)_T,V_* = 0, the following equilibrium equations [10] are obtained, respectively:

$$\begin{array}{l} \tilde{P}\tilde{V}/\tilde{T}={\left[1-2^{-1/6}y{\left(y\tilde{V}\right)}^{-1/3}\right]}^{-1}\\ \;+\left(2y/\tilde{T}\right){\left(y\tilde{V}\right)}^{-2}\left[1.011{\left(y\tilde{V}\right)}^{-2}-1.2045\right] \end{array}$$ (9)

$$\begin{array}{l} \left(s/3c\right)\left[\left(s-1\right)/s+y^{-1}\ell n\left(1-y\right)\right]\\ \;=\left[2^{-1/6}y{\left(y\tilde{V}\right)}^{-1/3}-1/3\right]{\left[1-2^{-1/6}y{\left(y\tilde{V}\right)}^{-1/3}\right]}^{-1}\\ \;+\left[y/\left(6\tilde{T}\right)\right]{\left(y\tilde{V}\right)}^{-2}\left[2.409-3.003{\left(y\tilde{V}\right)}^{-2}\right] \end{array}$$ (10)

where *y* = 1 − *h* is the fraction of occupied sites, and *s* and 3*c* are the number of segments per molecule and the external degrees of freedom per molecule, respectively. As in previous work, we take *s/3c* = 1. Note that the term *(s − 1)/s* in eq (10) approaches unity for large molecules.

A basic assumption sufficient for the application of the hole theory to our densification model is

$$dT_{g}^{†}/dP={\left(\partial T/\partial P\right)}_{h}.$$ (11)

Gee [15] has shown that such an equation is valid for a single ordering parameter which is frozen in the glass. However, since *h* has been found to vary slightly with temperature and pressure in the glass [16, 17, 18], eq (11) must be revaluated to assess its validity for the more general case.

Consider the single-valued function *V* = *V (T, P, h)* for which, by the usual definitions,

$$\begin{array}{l} -\kappa ={\left(\partial \ell nV/\partial P\right)}_{T}\\ \;={\left(\partial \ell nV/\partial P\right)}_{T,h}+{\left(\partial \ell nV/\partial h\right)}_{T,P}{\left(\partial h/\partial P\right)}_{T}\\ \alpha ={\left(\partial \ell nV/\partial T\right)}_{P}\\ \;={\left(\partial \ell nV/\partial T\right)}_{P,h}+{\left(\partial \ell nV/\partial h\right)}_{T,P}{\left(\partial h/\partial T\right)}_{P}. \end{array}$$

Since there are three independent variables (in the general case, the derivatives with two fixed arguments (suscripts) are the same for liquid and glass. (For the glass it is understood that these derivatives pertain to constant *P′* and *κ*.) The differences become

$$\begin{array}{l} -\Delta \kappa ={\left(\partial \ell nV/\partial h\right)}_{T,P}\left[{\left(\partial h/\partial P\right)}_{T,\ell }-{\left(\partial h/\partial P\right)}_{T,g}\right]\\ \Delta \alpha ={\left(\partial \ell nV/\partial h\right)}_{T,P}\left[{\left(\partial h/\partial T\right)}_{P,\ell }-{\left(\partial h/\partial T\right)}_{P,g}\right], \end{array}$$

where the subscripts *ℓ* and *g* again pertain to liquid and glass. Recalling that $dT_{g}^{†}/dP=\Delta k/\Delta \alpha$, the ratio of the above equations is

$$dT_{g}^{†}/dP={\left(\partial T/\partial P\right)}_{h,\ell }F_{P}/F_{T}$$ (12)

where the “freezing fractions” *F_P_* and *F_T_* are

$$\begin{array}{l} F_{P}=1-{\left(\partial h/\partial P\right)}_{T,g}/{\left(\partial h/\partial P\right)}_{T,\ell }\\ F_{T}=1-{\left(\partial h/\partial T\right)}_{P,g}/{\left(\partial h/\partial T\right)}_{P,\ell } \end{array}$$ (13)

as defined in ref. [14]. Note that when *F _T_ = F _P_*, eqs (11) and (12) coincide.

Since the variables *T, P*, and *h* are continuous at $T_{g}^{†}$, it follows that

$${\left(\partial T/\partial P\right)}_{h,\ell }={\left(\partial T/\partial P\right)}_{h,g}={\left(\partial T/\partial P\right)}_{h}$$

along this transition line. From partial differential equations, i.e.

$$\begin{array}{r} {\left(\partial T/\partial P\right)}_{h}=-{\left(\partial h/\partial P\right)}_{T,\ell }/{\left(\partial h/\partial T\right)}_{P,\ell }\\ =-{\left(\partial h/\partial P\right)}_{T,g}/{\left(\partial /h/\partial T\right)}_{T,g,} \end{array}$$

it follows that

$${\left(\partial h/\partial P\right)}_{T,g}/{\left(\partial h/\partial P\right)}_{T,\ell }={\left(\partial h/\partial T\right)}_{p,g}/{\left(\partial h/\partial T\right)}_{P,\ell ,}$$

which, as seen from eq (13), is tantamount to *F_T_ = F_p_*. Accordingly, since eqs (12) and (13) coincide, the validity of eq (12) is extended to a single ordering parameter which need not be “frozen” in the glass.

To our knowledge both of the above freezing fractions have been evaluated for only two systems, namely polyvinyl acetate) [16] and selenium [19]. According to the best analysis given in ref. [16], *F_P_* = 0.88 and *F_T_* = 0.82 for which the ratio *F_P_/F_T_* = 1.07, which corresponds to a 7 percent discrepancy in eq (11) for polyvinyl acetate). A similar conclusion follows for Se. Since the above analysis shows that *F_T_ = F_P_*, these differences are taken to be artifacts resulting from numerical inaccuracies.

The next step is the evaluation of ${\left(\partial \tilde{T}/\partial \tilde{P}\right)}_{h}$ at equilibrium. From simultaneous numerical solutions of eqs (9) and (10), values of *h* = 1 *− y* are obtained at a given set of reduced temperatures and pressures. For computational purposes it is convenient to replace ${\left(\partial \tilde{T}/\partial \tilde{P}\right)}_{h}$ by the ratio $-{\left(\partial h/\partial \tilde{P}\right)}_{\tilde{T}}/{\left(\partial h/\partial \tilde{T}\right)}_{P}$. With constant increments Δ *x(x = T* or P), it is easily shown for a quadratic dependence of *y* on *x* that

$${\left(dy/dx\right)}_{i}=\left(y_{i+1}-y_{i-1}\right)/\left(2\Delta x\right).$$

This procedure is used to generate a set of ${\left(\partial h/\partial \tilde{P}\right)}_{\tilde{T}}$ and ${\left(\partial h/\partial \tilde{T}\right)}_{P}$ values over the desired range from the sets of quadratic arcs defined by three adjacent points. Using a least squares fit, the approximation

$${\left(\partial \tilde{T}/\partial \tilde{P}\right)}_{h}=0.00502+0.198\tilde{T}+31.476{\tilde{T}}^{2}$$ (14)

is found to be accurate within a residual standard deviation of 0.2 percent at atmospheric pressure over the range 0.01 ≤ $\tilde{T}$ ≤ 0.04. From eqs (8) and (11)

$$dT_{g}^{†}/dP=T*{\left(\partial \tilde{T}/\partial \tilde{P}\right)}_{h}/P*.$$ (15)

Substitution of eq (15) into eq (5b) gives the desired relation for the densification rate,

$$\kappa^{\prime }=\Delta \alpha \left[T*{\left(\partial \tilde{T}/\partial \tilde{P}\right)}_{h}/P*-dT_{g}/dP\right],$$ (16)

where all quantities are evaluated at *T = T_g_*.

Equation (16) may be rewritten (see for example eq (14) in Ref. [14]) as

$$\kappa^{\prime }=-\Delta \alpha \left[{\left(\partial T/\partial h\right)}_{P}\times dh/dP\right]$$ (16′)

where the total derivative on the right hand side is to be taken along the *T_g_(P)* line. Provided the pressure coefficient of *T_g_* has been determined with sufficient accuracy, there apparently is no numerical advantage in using eq (16′).

It is mentioned in the last section that eq (7) appears to be a good approximation for most polymers. Assuming this relation, we may estimate *κ′* from volume-temperature and heat capacity data using the relation

$$\kappa^{\prime }=\Delta \alpha \left[T*{\left(\partial T/\partial P\right)}_{h}/P*-T_{g}V\Delta \alpha /\Delta C_{P}\right].$$ (17)

Olabisi and Simha [17] have shown for most polymers studied by them that the scaling factor P* may be determined from the other two by means of the empirical relation

$$P*=\left(T*/V*\right)\exp\left(1.319-1.493\times 10^{-4}T*\right)$$ (18)

where the dimensions are K, bar, and em^3^/g. Thus it appears feasible to estimate *κ′* from appropriate data at atmospheric pressure only. This possibility is tested later in this paper.

According to Wunderlich [20] it is possible to estimate Δ *C_P_ T_g_* to within about ±2J/(mol − K) by applying the “ride of constant Δ *C_P_*” The molecular repeat units are broken up into fundamental units or “beads” which loosen up in the *T_g_* process. Each bead is assigned the value 11.3 J/(mol − K). The contributions of the beads to Δ *C_P_* are assumed to be additive. Accepting the validity of this rule, it appears possible to obtain a crude estimate of *κ′* from hole theory applied to volume-temperature measurements alone.

## 4. Results

### 4.1. Data Sources

Although we refer usually to the original sources, there are collections of data on the pertinent quantities in the literature, which are sometimes cited here. Extensive lists of polymers and their values of *T_g_* appear in refs. [21–23], where the last is restricted to fluorine-containing systems. Tables of *T_g_* and Δ *α* are included in Refs. [24–26], and *T_g_* and Δ *C_P_* in Refs. [27] and [28]. Reference [29], which is occasionally cited here, contains a more critical evaluation of *C_p_* data on polymers for which the values on the same substance are often based on averages from different sources over wide ranges of temperature. Lists of polymers and their scaling factors based on the hole theory appear in Refs. [17] and [30]; however, P* is not available in the latter. Pyrolysis data on polymers are contained in refs. [31–34]. These are useful to prevent degradation during the densification process and to optimize the amount of densification. Finally, ref. [35] gives an extensive list of refractive indices for polymers.

The number of digits for the values given in the subsequent tables is not intended to be an indication of precision or accuracy. Usually these numbers correspond to those given by the data sources. It is our opinion that most of the entries in these tables have more digits than can be justified as significant.

Table 1 gives the lists of polymers studied, abbreviations used here, and their values of *T_g_*. In all tables the sequence is in order of increasing *T_g_*.

### 4.2. Scaling Factors

Table 2 gives the scaling factors based on the hole theory of Simha and Somcynsky [10]. These are determined through superposition of experimental equilibrium data on each polymer with respect to the theoretical equation of state. In this work the scaling factors are used solely to estimate $dT_{g}^{†}/dP=\Delta \kappa /\Delta \alpha$ for each polymer using eq (15). When two numbers appear in the reference column (in table 2 only), the first applies to the data source, and the second to the work by which the scaling factors are evaluated. When only one number appears, the scaling factors are either evaluated in the reference given, or by us.

Two values of P* for each polymer (or row) usually appear. The first of these (P*) is determined in the usual way through superposition as mentioned above. The second $P_{\text{cala}}^{*}$ is obtained from eq (18). When volume-temperature data are available at atmospheric pressure only, it is necessary to use eq (18) to estimate P*. With the exception of PDMSi, P* and $P_{\text{cala}}^{*}$ agree to within 17 percent with an 8 percent relative standard deviation of differences over 17 pairs. With PDMSi the disparity of 87 percent is outstanding, and it is to be noted that the reduced glass temperature lies significantly outside the range for which eq (18) was deduced. Similarly, V* and T* are obtained at considerably higher temperatures than those employed here. A decrease of T* by 7.2 percent and a concomitant decrease of V* by 2.4 percent over 100 K has been estimated [30] for this polymer. Accordingly, the scaling factors cannot be assigned significant constant values over the experimental range.

In order to obtain some measure of the uncertainty in the scaling factors, several data sources on each polymer are sometimes included.

### 4.3. Densification Rates from *PVT* Data

Table 3 gives the results of calculations of the densification rates from *PVT* data without recourse to molecular theory. $\kappa_{1}^{\prime }$ is determined from the definition:

$$\kappa_{1}^{\prime }=\left(V_{A}-V_{B}\right)/V_{A}P^{\prime }$$ (4′)

which is identical to eq (4) setting Δ *P* = *P*′ except that *V_A_* replaced *V*_0_. The difference between the values of $\kappa_{1}^{\prime }$ determined from eqs (4) and (4′) are insignificant in comparison with experimental uncertainty, $\kappa_{2}^{\prime }$ is determined from eq (5b). Note that there are only two polymers, PVAc and P*α*MS for which we found sufficient information to determine both $\kappa_{1}^{\prime }$ and $\kappa_{2}^{\prime }$ Although the two methods are not necessarily fully equivalent because of the assumptions used to derive eqs (5), the agreement in both cases is good. In instances of more than one set of values per polymer, it is clear that the deviations in $dT_{g}^{†}/dP$ have the largest effect on the uncertainty of $\kappa_{2}^{\prime }$. These apparent discrepancies are usually consistent with the differences in Δ κ. With polystyrene the maximum deviation in $dT_{g}^{†}/dP$ is 38 percent compared with those for *dT_g_/dP*, 23 percent and Δ α, 10 percent. Since $\kappa_{2}^{\prime }$ involves the difference between the two transition rates, its maximum deviation is magnified to 56 percent with its relative standard deviation over the five values being 26 percent. It is interesting to note that the direct method giving $\kappa_{1}^{\prime }$ which one might expect to be more reliable, yields values for which the maximum deviation (for polystyrene) is 86 percent with relative standard deviation over seven values being 35 percent. The ratio of average values, ${\bar{\kappa }}_{2}^{\prime }/{\bar{\kappa }}_{1}^{\prime }$, is 1.6. These discrepancies are a measure of the difficulties in obtaining reliable *PVT* data on glassy polymers.

In many instances the values of Δ α at *T_g_* and the required transition rates are not tabulated in the data sources and, therefore, had to be evaluated. The accuracy of these evaluations may be considerably limited when the data are presented in graphical form only. In ref. [47] the values of Δκ are determined by a different definition from the one used by us. In our definition *K_g_* is taken to be an isochronal (non-relaxing) function of temperature and pressure and therefore must be derived from data at temperatures below (or pressures above) the glass transition region. Δ κ at *T_g_* is then obtained by extrapolation. This, apparently, was not done by Hellwege et al. [47], at least over the appropriate temperature range for the data to be effectively isochronal. The distinction between the two definitions is clearly illustrated by Boyer [57]. Our larger values of Δ κ are determined from the Tait parameters given in ref. [53], which apply to the data of Hellwege et al. [47]. Note that the values of $dT_{g}^{†}/dP$ from reevaluating [53] their data are in good agreement with most of the others on the same polymers except for PMMA. This discrepancy would be increased by using their value of Δ κ given in ref. [47], along with poorer overall agreement with the other two polymers.

In ref. [1] the transition rates are given as tangent values along the transition lines at each experimental pressure. Here, we use the secant values *dT_g_/dP* and $dT_{g}^{†}/dP$ between 0 and 800 bar. This procedure gives average values and is more consistent with other treatments.

The smallest value of *κ′* = 0.7 Mbar^−1^ in table 3 applies to PnBMA. Such a small value implies that the first Ehrenfest equation is a good approximation for this polymer. [See eqs (5c) and (6b)].

### 4.4. Theoretical Estimation of the Transition Rate

$$dT_{g}^{†}/dP$$

As stated in section 3 the transition rate $dT_{g}^{†}/dP=\Delta \kappa /\Delta \alpha$ applicable to the constant formation history, may be estimated from the value of *(∂T/∂P)_h_* at *T_g_*. Table 4 summarizes the results of these calculations. In all cases except for PDMSi and PIB the data encompass *T_g_*. With these two polymers the first reference for each applies to the source of data at atmospheric pressure, and the second, at elevated pressures.

Equation (14) and the scaling temperatures and pressures as applied to eq (15) provide the requisite information. The distinction between the values of *(∂T/∂P)_h_* in columns A and B is that they correspond to P* and $P_{\text{cacl}}^{*}$ respectively, in table 2. Values of $dT_{g}^{†}/dP$ are included for comparison with those of (*∂T/∂P*)*_h_* in cases where there is sufficient experimental information.

With natural rubber (NR) Δ κ is determined from dynamic compressibility data [60]. This involves the measurement of the adiabatic compressibility in a hydrostatic stress field alternating at low audio frequencies. The low- and high frequency limiting compressibilities are converted from adiabatic to isothermal conditions, providing the difference Δ κ. The fact that Δ κ is determined at about 20 K above normal *T_g_* is expected to have no appreciable effect.

Except for PDMSi, the corresponding values of (*∂T/∂P*)*_h_* are nearly the same in columns A and B. The discrepancy for PDMSi is explained by the factors mentioned earlier. (See sec. 4.2.). Excluding this polymer, the relative standard deviation of the differences between corresponding values in these columns is 6 percent, which is considered to be good agreement. For comparison with experiment, the residual standard deviation between corresponding values of (*∂T/∂P*)*_h_* (column A) and $dT_{g}^{†}/dP$ is 18 percent. Poor agreement is noted for PnMBa, and two samples of *a* -PMMA.

Over a single substance, for example polystyrene where we have four sets of values, the relative standard deviations with respect to the averages for (*∂T/∂P*)*_h_* (column A) and $dT_{g}^{†}/dP$ are 6 and 15 percent, respectively. That for the differences between corresponding values of these quantities is 11 percent. Thus, based on these simple statistics, the most serious limitation is not the inadequacy of the theory, but the uncertainty in the experimental determination of $dT_{g}^{†}/dP$. The agreement between values of $dT_{g}^{†}/dP$ from different investigators is even worse for *a*-PMMA.

### 4.5. Application of Hole Theory

After determining the values of (*∂T/∂P*)*_h_* for each substance, *κ′* may be estimated by eq (16), where *dT_g_/dP* may be determined by means of *PVT* data, dynamic measurements at elevated pressures, or heat capacity and thermal expansion data, both at atmospheric pressure.

#### PVT Data

Since *PVT* measurements are often made by the variable formation history only (for example, polypropylene, ref. [40]), there is insufficient information to determine Δ κ, and hence $dT_{g}^{†}/dP$ to be applied to eq (5b). Accordingly, this quantity is replaced by $T*{\left(\partial \tilde{T}/\partial \tilde{P}\right)}_{h}/P*$ leading to eq (16). The results of these estimates are given in table 5, where κ′ corresponds to *κ′*_2_ in table 3. In all cases (*∂T/∂P*)*_h_* is taken from column A of table 4. Since the expression for *κ′* involves the difference between two transition rates, its value is very sensitive to (*∂T/∂P*)*_h_*. This effect is reflected in the large standard deviation, 2.5 Mbar^−1^, with a relative value of 28 percent, for the differences over 14 pairs of corresponding values of *κ′*_2_ and κ*'* in tables 3 and 5. The values of P*α*MS are not included in this calculation because there are insufficient data in ref. [56] to determine the scaling factors applicable to this particular sample. Based on the fact that the standard deviation of *κ′*_2_ for polystyrene (table 3) over five values is 2.8 Mbar^−1^ corresponding to 26 percent, the overall 28 percent value above appears to be dominated by experimental uncertainty.

#### Dynamic Data

From the assumption that the value of *dT_g_/dP*, approximates that of (*∂T/∂P*)*_ω_*, where *ω* is the angular frequency, frequency-temperature-pressure superposition of dynamic data, including dielectric and ultrasonic, may be used to determine *dT_g_/dP* appearing in eq (16). The validity of this assumption is, of course, subject to the condition that (*∂T/∂P*)*_ω_* for the *T_g_* -process is essentially independent of frequency. (Numerical comparisons between different experimental transition rates are made below.)

The results of these calculations are given in table 6. Since values of (*∂T/∂P*)*_h_* in column A of table 4 involve fewer assumptions than those in column B, the former are used where there is a choice. The standard deviation of the differences of κ*′* over eight pairs, where there are values on the same substances in table 3 including *κ′*_1_ for Pcarb, is 2.2 Mbar^−1^ or 30 percent, which is about the same as the experimental uncertainty given above (2.8 Mbar^−1^ or 26 percent) for polystyrene. This value is also about the same as the 28 percent value given for the *PVT* data even though data on different substances are involved. It is possible, however, that *dT_g_/dP* values determined from dynamic data, in particular dielectric, where high resolution is obtained, are more reliable than *PVT* values. *T_g_* determinations from *PVT* data usually involve extrapolations which are not used in the superposition of dynamic data.

#### Heat Capacity and Thermal Expansion

The estimation of *κ′* from heat capacity and thermal expansion data is based on the apparent validity of the second Ehrenfest equation [eq (7)]. (This relation is tested in the next section.) The results of the calculations based on eq (17) are summarized in table 7. In this case the statistics may not be meaningful because there are only five values of *κ′* which correspond to those in table 3 including *κ*_1_ for Pcarb. P*α*MS is excluded for the reason given above. The standard deviation of the differences is 1.4 Mbar^−1^ or 17 percent, which is somewhat less than the experimental uncertainty (26 percent) based on polysytrene data (table 3). In view of the high experimental uncertainty for all methods, this method of estimating *κ′* appears to be reliable, except for PDMSi and PoMS.

In ref. [12] a negative value of *κ′* for PDMSi [based on eq (17)] is reported. This is a surprising, but not necessarily an incorrect result. The analysis of this polymer is hindered by the lack of good thermal expansion data through *T_g_*, largely a consequence of the low temperatures required, and the strong tendency for this polymer to crystallize. The negative value of *κ′* is obtained by using the value of Act = 10.28 K^−1^ from table I of ref. [26]. This value is based on the linear thermal expansion data of Weir, Leser, and Wood [58]. After a thorough examination of their results and consultation with Dr. Wood, it was decided that the temperature range for which *Vα_l_* was evaluated is too small and too remote from *T_g_* to evaluate Δ *α* at *T_g_*. In order to obtain what we consider to be the best available estimate of Act, we used the value of *Vα_g_* = 2.7 × 10^−4^ cm^3^/(g-K) from ref. [58], and *α_l_* = 8.7 × 10^−4^ K^−1^ and *V_g_* = 0.904 cm^3^/g from the density-temperature equation of Shih and Flory [36]. Although this equation is derived from data at temperatures well above *T_g_*, its nearly linear response apparently allows valid extrapolation to much lower temperatures. The value *V_g_a_l_* = 7.9 × 10^−4^ cm^3^/(g-K) is slightly less than the average, 8.7 × 10^−4^, of the others for this polymer in table I of ref. [26] which are obtained from different sources of data at higher temperatures not encompassing *T_g_*. Also the extrapolated value of *V_g_* = 0.904 cm^3^/g above essentially coincides with 0.905 in ref. [26]. The revised thermal expansion values give the positive value of *κ′* shown in table 7.

### 4.6. Comparison of Experimental Transition Rates

In tables 6 and 7 the assumptions that *dT_g_/dP* could be replaced by (*∂T/∂P*)*ω* or TVΔ α/Δ *C_p_*, respectively, are employed. In table 8 values of these quantities are compared for each polymer. A similar table was prepared by O'Reilly [13] in 1962 for glass-forming liquids not restricted to polymers. Values of Δ κ/Δ α are also included here for comparison; however, agreement with *dTg/dP* is not expected since the validity of the inequality

$$dT_{g}/dP<\Delta \kappa /\Delta \alpha$$

appears to be quite strong and general. In most instances, agreement between *dT_g_/dP, (∂T/∂P)_ω_*, and *TV*α*/*Δ *C_P_* seems to be within experimental error. Small differences may be anticipated because the conditions under which these quantities are evaluated may be vastly different.

According to these results the Prigogine-Defay ratio

$$r=\Delta \kappa \Delta C_{P}/\left[TV{\left(\Delta \alpha \right)}^{2}\right]$$

is essentially unity for natural rubber and PaMS. Unfortunately, we do not have a *PVT* value of *dT_g_/dP* for the former to test the validity of the Ehrenfest equations [eqs (6) and (7)]. With P*α*MS it would appear that although neither of the Ehrenfest equations is obeyed, the Prigogine-Defay ratio is still unity, which is an atypical result. This implies that $dT_{g}^{†}/dP$ for volume and entropy are equal, but *dT_g_/dP* is distinct. However, since data in tabular, or even graphical form, are not included in ref. [56], evaluation of these numbers cannot be scrutinized. Poor agreement for PVC in all cases is apparent; however, this may be a result of sample differences including the degree of crystallinity which is difficult to control in this polymer. Also poor agreement is noted for *a*-PMMA of ref. [9], where both quantities are obtained from the same sample. On the other hand, the data in the row above on the same polymer reveal good agreement including that with *dT_g_/dP* (PVT) of ref. [9]. In all cases agreement is very good for polystyrene.

These results indicate that *dT_g_/dP* = (*∂T/∂P*)*_ω_* is a valid relation and *dT_g_/dP = TV* Δ*α/*Δ*CP* seems to hold most of the time. The validity of the first may be argued on a qualitative phenomenological basis (see sec. 2.) The second relation is evaluated at atmospheric pressure only. There is no apparent reason to assume that the approximation will be as good at elevated pressures.

In section 4.3 we mentioned that *PVT* data on PnBMA suggest that the first Ehrenfest equation [eq (6b)] is a good approximation for this polymer. This result is tantamount to essentially no densification. (See table 3.) Unfortunately, we have no heat capacity values for this polymer, which are needed to check the second Ehrenfest equation [eq (7)]. In section 3 we noted the possibility of using the “rule of constant Δ*Cp*” [20] to estimate the heat capacity difference at *T_g_*. (For a comparison of experimental and “bead” values of ΔC*_P_* on polymers, see ref. [28].) For PnBMA the molecular weight of the polymeric repeat unit is 142.2 g/mol. Assigning one bead to each of the two carbon backbones, and one to the oxygen atom, we obtain a total of three beads, which for 11.3 J/(mol-K-bead) gives Δ*C_P_* = 0.24 J/(g-K). Taking this value along with those for Act and *T_g_* from tables 3 and 4, respectively, and *V_g_* = 0.946 cm^3^/g from ref. [43], we obtain *TV*Δ*α/* Δ*Cp* = 20 K/kbar, which is in good agreement with 20.4 in table 3. Thus both of the Ehrenfest equations appear to be fairly good approximations for this polymer, along with a corresponding Prigogine-Defay ratio of nearly unity. (The value 1.2 is obtained for PnMBA. The average value obtained from table 8, exclusive of NR and P*α*MS, which were treated separately, is 2.1.) These results imply that both the density and entropy of PnBMA are essentially independent of formation pressure, at least at low pressures.

### 4.7. Pressure Dependence of *κ′* and Limitations Imposed by Chemical Instability

The previous discussions in this paper pertain to the initial values of *κ'* or at least at very low formation pressures. Most of these are either tangent values at *P* = 0 (atmospheric pressure) or secant values obtained from *P*′ = 1 kbar or less. There are data in the literature, however, which include densifications obtained at different formation pressures.

**Figure 4:**
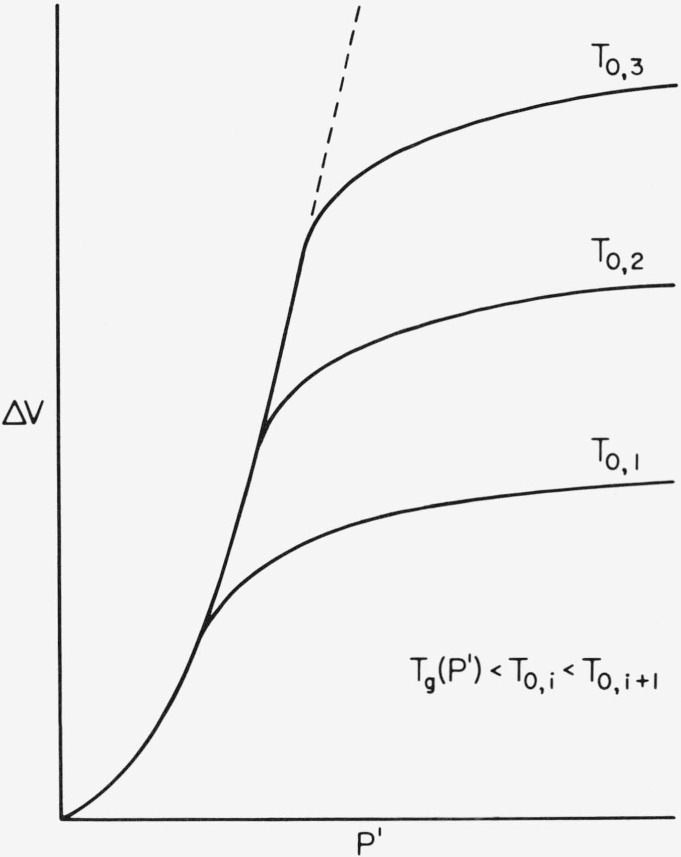
Illustration of the influence of the pressurization temperature T_0_ on the densification process. The dashed line is the envelope approached at large temperatures.

There are two important physical considerations in optimizing the procedure to obtain “permanent,” densified glasses. The first and more obvious, is to select and maintain the temperature of depressurization at temperatures sufficiently below *T_g_*. It is clear that the ambient conditions must be such as to maintain structural relaxation times which are large in comparison to the desired “lifetime” of the glass. Accordingly, high *T_g_* substances are preferable for room temperature stability. The second is to choose *T*_0_, the temperature of isothermal pressurization, large enough that the equilibrium melt is always maintained during pressurization. Stated alternatively, the inequality

$$T_{0}>T_{g}\left(P^{\prime }\right)$$ (19)

must be approximately satisfied, as illustrated in figure 1. This condition implies that the effective time of the pressurization process must be large in comparison to the structural relaxation time at the final pressure *P′*. In cases where *T*_0_ < *Tg*(*P′)* there will be a much smaller contribution to the densification process when the condition *T_0_ = T_g_(P)* is approximated and exceeded during pressurization. This situation is revealed by a leveling off in the volume as illustrated schematically in figure 4, where volume changes are plotted with respect to formation pressure at different pressurization temperatures. The densification is expected to be independent of *T*_0_ at low pressures, when in eq (19) is satisfied, as is revealed by the coalescence of these curves with decreasing *P′*. Such a coalescence is not expected, however, when *T*_0_ < *T_g_* as is illustrated by the data of Shishkin [54] on polystyrene. In figure 4 the dashed line represents the extension of the envelope established from arbitrarily large values of *T*_0_.

One of the better experimental examples which illustrates the behavior shown in figure 4 is provided by the data of Shishkin on PMMA and PS. Formation pressures up to four kbar are applied; but not all of the pressurization temperatures are above *T_g_*(*P*′). At the lower pressures, *κ'* increases with *P*′ as is indicated by the increasing slopes of Shishkin's volume-formation pressure curves, and as is shown in figure 4. This is the opposite of the trend for the isothermal compressibility κ, which decreases with increasing pressure (see for example refs. [12] and [74]). The data of Shishkin, as well as those of Kirmmel and Uhlmann [75] on PMMA, show that some densification is possible with pressurization temperatures below *T_g_*, but the effect is diminished as the difference between these two temperatures is increased. Other examples illustrating the dependence of densification on formation pressure are refs. [6], [9] and [55] on polystyrene, and [56] on P*α*MS. Yourtee and Cooper [6] observe a very slight decrease in the densification rate with formation pressure for polystyrene over a 6 kbar range. For the same polymer, Weitz and Wunderlich [9] find a much larger dependence with the same trend, where the density gradually becomes nearly constant at 4 kbar. These trends are contrary to the marked increase in the densification rate with formation pressure observed by Shishkin on PS and PMMA and Iehihara et al. [56] on P*α*MS. *κ′* does not necessarily have to tend to zero for the volume to be non-negative at large formation pressures. Using our definition of κ′, the densified volume tends to zero at arbitrarily large formation pressures when *κ′* is a positive constant.

According to the experiments of Weitz and Wunderlich on polystyrene, there is a monotonic increase in density at a decreasing rate which the density seems to level off at 4 kbar. Thus, beyond this point the formation pressure would have no effect on the densification process. On the other hand, with most of the other investigations mentioned above, including those on polystyrene, it would appear that chemical stability is the limiting factor. Whether the reaction rate constant of a given rate process increases or decreases with pressure depends upon the sign of its corresponding activation volume [76]. In most cases it is expected that the total activation volume will be positive with a corresponding increase in the effective decomposition temperature with increasing pressure. This behavior may be complicated, however, by the different temperature and pressure dependencies of the various decomposition modes, and, possibly by the initiation of new ones at elevated pressures.

The important consideration here is whether, or not, the decomposition temperature and *T_g_* -pressure curves come sufficiently close at any point to limit the densification process. For example, with polytetrafluoroethylene the increase of decomposition temperature with pressure is only about 3.5 K/kbar [77]. Although this rate is small, the decomposition temperature is sufficiently remote from the observed phase transitions, since its initial (atmospheric pressure) value is about 700°C. In addition, the melting and decomposition curves diverge with increasing pressure over the experimental range of 28 kbar, investigated so far.

**Figure 5:**
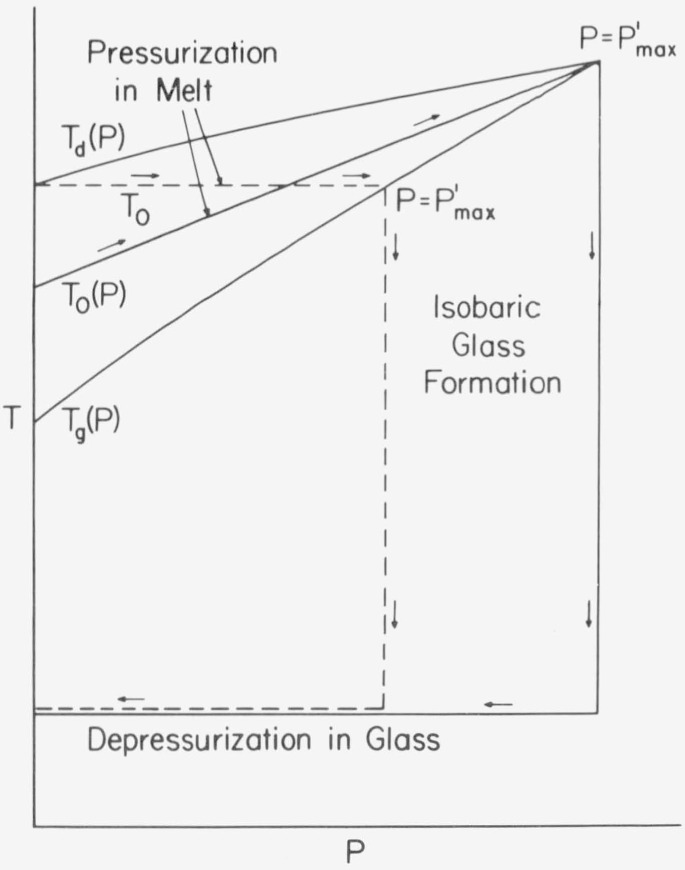
Schematic illustration of temperature-pressure history used to optimize the densification process before the onset of pyrolysis.

In cases where the decomposition temperature *T_d_* increases with pressure, *T*_0_ should also be allowed to increase with pressure to optimize the densification. With polytetrafluoroethylene this process would appear to continue without bound because of the observed divergence mentioned above. In instances where *T_d_* and *T_g_* converge or intersect at a finite pressure, the densification would be essentially limited b) the effective intersection temperature as illustrated in figure 5. Except for the polymer mentioned above, pressure dependent pyrolysis data are apparently non-existent in the literature^4^.

The results in table 9 summarize an attempt to estimate the optimum densification on a few polymers beyond which thermal decomposition would occur. In the absence of reliable pyrolysis data at elevated pressures, we will estimate optimum densification by commencing isothermal pressurization at *T*_0_ *= T_d_*. Since in most cases *T_d_* is expected to increase with pressure, this procedure should underestimate the maximum densification as illustrated by the lower value of P′_max_ obtained by the dashed line path in figure 5.

In table 9 *T**_d_* is taken arbitrarily at the value for which the initial reaction rate constant k = 1%/hr, applicable to the total degredation process. Assuming Arrhenius behavior *T_d_* may be calculated from the relation

$$1/T_{d}=1/T-\left(R/\Delta H\right)\ell n\left[\left(1/60\right)/k\left(T\right)\right]$$

where *R* = 8.314 J/(mol-K) and Δ*H* is the activation energy. *T* is arbitrarly chosen from the closest data point to *T_d_*, which in all cases, but one (PVAc), involves extrapolation. These decomposition temperatures correspond to those given in table 7 of ref. [34], except the latter apparently apply to *k* = 1 percent/min and, accordingly, are larger. The ceiling temperatures in the same table, which apply to the propagation mode at equilibrium, are apparently not relevant to the densification process. *T_g_, dT_g_/dP*, and *κ′* are selected from previous tables in this paper. *P′_max_*, the pressure corresponding to the onset of pyrolysis at *T_0_* = *T* & and − (Δ*V*/*V*)_max_, the corresponding maximum densification, are obtained from the simple relations

$$\begin{array}{l} \;{P^{\prime }}_{\max}=\left[T_{d}-T_{g}\left(0\right)\right]/\left(dT_{g}/dP\right)\\ -{\left(\Delta V/V\right)}_{\max}=\kappa^{\prime }\times {P^{\prime }}_{\max}. \end{array}$$

From these results it appears that *P'*_max_ varies inversely with *T_g_;* however, no trend is apparent for (ΔV/V)max.

### 4.8. Dependence of Refractive Index on Densification

A reliable estimate of the change of refractive index on densification should be obtained by means of the Lorentz-Lorenz equation,

$$\left(n^{2}-1\right)/\left(n^{2}+2\right)=K\rho ,$$ (20)

where *n* is the index of refraction and *p* the density. *K* depends upon the polarizability, which is expected to be essentially independent of formation pressure, or alternatively, the density at constant temperature and pressure. The relative change of index of refraction with formation pressure,

$$\delta^{\prime }=\left(1/n\right){\left(\partial n/\partial P\right)}_{T,P,}$$

is obtained explicitly by differentiation of eq (20), viz,

$$\delta^{\prime }=\left(1/6n^{2}\right)\left(n^{2}-1\right)\left(n^{2}+2\right)\kappa^{\prime }.$$ (21)

Table 10 presents the results for polymers for which values for *n_D_* (sodium *D* line) are available from ref. [35] with *κ'* selected from our tables. The *n_D_* values are converted to those at *T_g_* by means of the temperature coefficients given in ref. [84]. As seen from the table, these corrections are insignificant. Since all of these values range between 1.48 and 1.58, a very slight (10 percent) error will be incorporated in δ*′* by taking the function *f(n)* = *(l/6n^2^)(n^2^ − l)(n^2^* + 2) as a constant, as revealed by the table. Accordingly, in view of the large experimental uncertainties in *κ′* (35 percent for polystyrene), the additional uncertainties obtained on replacing eq (21) by the approximation

$$\delta^{\prime }=0.4\kappa^{\prime }$$

are slight. The values of *8'* in the table however, are calculated from eq (21). We do not have any direct experimental data giving the dependence of the index of refraction on formation pressure.

These evaluations have potential application in optimization or adjustment of the refractive indices of plastic lenses by appropriately setting the molding pressure. The values in the last column in table 10 give the relative percent changes (Δ*n/n*) resulting from a moding pressure of 10 kbar. For PS and PMMA, which are common constituents for plastic lenses, *n* would change by 4 and 3 percent, respectively. However, it was estimated in the last section that thermal decomposition of these polymers would limit the pressurization to 7 and 8 kbar, respectively. In these analyses isothermal pressurization is considered at the decomposition temperature. If this temperature increases with pressure as indicated by ref. [78] for PS and PMMA, an additional increase in their refractive indices could be obtained by appropriately increasing the temperature during pressurization.

## 5. Conclusion

Several methods have been evaluated to estimate the densification rate, *κ′* applicable to glass formation by isobaric cooling at constant rate. Other than the direct measurement of the volume difference in the glass, *κ′* is always computed from an expression involving the difference between two transition rates, *dT_g_/dP* and $dT_{g}^{†}/dP$. The hole theory is shown to be sufficiently accurate in estimating $dT_{g}^{†}/dP$ for the 23 polymers evaluated except for possibly those of dimethyl siloxane and α-methyl styrene. With these it is not clear whether the discrepancies result from experimental error or lack of generality in the application of the theory. Although *dT_g_/dP* is only evaluated experimentally, there are independent alternatives. The simplest of these involves the differences between thermal expansions and heat capacities at *T_g_* for liquid and glass at atmospheric pressure only.

The principal problem in the estimation of densification using these procedures appears to be the large amount of experimental uncertainty in all of the relevant quantities, in particular, the compressibility. Since the expression for *κ′* involves the difference between two quantities of similar magnitude, even small experimental errors may have a pronounced effect. Accordingly, it is difficult to assess the relative merits of the different methods employed here, including the application of the hole theory.

The results of these analyses appear to have practical applications. Densifying glasses produces a hardening effect as revealed by an increase in moduli. However, these effects do not appear to be as pronounced, in particular viscosity or relaxation time, as those obtained at the same volume, temperature, and pressure in the glass by commensurately decreasing the cooling rate at atmospheric pressure. This procedure however is usually not practical because of the large times required for glass formation. According to one investigation it is possible to optimize the ultimate properties through the appropriate adjustment of the formation or molding pressure. More work is necessary to establish the generality of this result and to determine the formation pressures for maximum yield stress. Moreover, the relation between the refractive index and densification quantity presented could be used to quantitatively regulate the refractive index of lenses through appropriate adjustment of the molding pressure. The maximum value would appear to be limited by chemical instability at the high temperatures necessary to exceed *T_g_(P)*, which increases with pressure. The simple relation given does not include the influence of densification on optical dispersion. Again, experimental work is required to assess the validity of our estimates and the possible influence of densification on dispersion.

## Figures and Tables

**Table 1:** List of polymers studied, abbreviations, and glass temperatures

Abbreviations	Polymer of	T*_g_*(K)
		
PDMSi	Dimethyl siloxane	150
PIB	Isobutylene	202
NR	Natural Rubber	204
PP	Propylene	244
SBR^a^	Styrene-butadiene	257
PMA	Methyl acrylate	282
PnBMA	*n*-butyl methacrylate	293
PVAc	Vinyl acetate	304
*i*-PMMA	Isotatie methyl methacrylate	320
PEMA	Ethyl metacrylate	337
PET	Ethylene terephthalate	340
PVC	Vinyl chloride	349
P4POS	4-phenoxystyrene	352
P3CS	3-chlorostyrene	363
PS	Styrene	363
*α*-PMMA	Atatic methyl methacrylate	378
PCHMA	Cyclohexyl methacrylate	380
P4M0S	4-methoxy styrene	381
P4CS	4-chlorostyrene	400
PoMS	*o*-methyl styrene	404
Pcarb	Carbonate of bis(phenol A)	416
P*α*MS (1)	*α*-methyl styrene (67% syndiotatic)	448
P*α*MS (2)	*α*-methyl styrene (95% syndiotatic)	455

a 55 percent Styrene.

**Table 2:** Polymer scaling factors

Polymer	Ref.	T*K	V* cm^3^/g	P* bar	P*calc 1 bar	ΔP*/P%
						
PDMSi	36, 30a	7893	0.9602	5061	9461	87
PDMSi	37	7893^b^	.9602^b^	5061	9461	87
PIB	38, 30a	11220	1.0902	–	7208	–
PIB	37	11220^b^	1.0902^b^	7316	7208	−1.4
NR	39	8344	1.0353	–	8672	–
PP	40	8966	1.1230	8437	7829	−7.2
SBR^c^	41	9800	0.9892	–	8577	–
PMA	42, 17	9200	.7925	–	10990	–
PnBMA	43, 17	9988	.9299	8456	9402	11
PVAc	1, 14	9419	.8141	9380	10600	13
PVAc	37	9419^b^	.8141^b^	9158	10600	16
*i*-PMMA	44	11170	.8160	10090	9659	−4.3
PEMA	45, 30a	11540	.8862	–	8694	–
PET	46	10870	.7406	–	10830	–
PVC	47d, 17	11320	.7105	10350	10990	6.2
PVC	48	11363	.7083	9783	11000	12
PS	15	12880	.9601	6628	7333	11
PS	47d, 17	12700	.9625	7638	7409	−2.9
PS	49	11630	.9480	7881	8082	2.6
PS	50	12680	.9598	7453	7440	−0.2
*a*-PMMA	48	11490	.8220	8987	9403	4.6
*a*-PMMA	43, 17	11920	.8370	9147	8984	−1.8
*a*-PMMA	47d, 17	11890	.8350	9303	9024	−3.0
PCHMA	43, 17	11290	0.8906	8382	8786	4.8
PoMS	50	12740	.9762	7458	7285	−2.3
Pcarb	51, 30a	12130	.8100	–	9156	–
PaMS (1)	52, 30a	12700	.9152	–	7792	–
PaMS (2)	52, 30a	12790	.8433	–	8403	–

a T* and V* only are determined in this reference.

b Volume-temperature data at atmospheric pressure taken from previous listing.

c 55 per cent Styrene.

d For additional comments of interpretation on these experimental data and evaluation of Tait parameters, see Ref. [53].

**Table 3:** Densification rate calculations at atmospheric pressure from PVT

Polymer	Ref.	Δα × 10^4^ K^−1^	*dT_g_/dP* K/kbar	*dT_g_*†*/dP* K/kbar	κ′_1_ Mhar^−1^	κ′**_2_** Mbar^−1^
						
PnBMA	43	1.69	20.4	24.3	–	0.7
PVAc	1	4.32	21.2	42.4	8.8	9.2
t-PMMA	44	3.49	21.1	35.2	–	4.9
PVC	47	2.93	13.5	35.5^a^–	–	6.4
PVC	48	3.71	14	46	–	12
PVC	2	–	–	–	4.4	–
P4P0S	5	–	–	–	7.2	–
P3CS	5	–	–	–	5.8	–
PS	15	2.84	31	71	–	11
PS	47	3.05	30.0	56.4^a^–	–	8.1
PS	49	3.15	25.0	49.8	–	7.8
PS	50	2.97	31.6	74.2	–	13
PS	2	–	–	–	5.5	–
PS	4	–	–	–	10	–
PS	5	–	–	–	7.2	–
PS	6	–	–	–	4.3	–
PS	8	–	–	–	5.6	–
PS	9	–	–	–	9.5	–
PS	54	–	–	–	4.5	–
PS	55	3.12	32	73	–	13.8
α-PMMA	48	3.1	18	71	–	15
α-PMMA	47	2.95	23	35.9^a^–	–	3.8
α-PMMA	43	2.35	23.6	54.5	–	7.3
α-PMMA	2	–	–	–	5.9	–
α-PMMA	54	–	–	–	4.9	–
PCHMA	43	3.38	22.4	59.8	–	13
P4MOS	5	–	–	–	7.1	–
P4CS	5	–	–	–	9.5	–
PoMS	50	2.71	34.2	73.0	–	11
Pcarb	2	–	–	–	5.1	–
PαMS^b^–	56	3.98	39	52	4.9	5.2

a The values of Δ *κ* here are not consistent with those given in Ref. [47]. For distinction, see text and/or Ref. [57].

b Tacticity not known to correspond to listings in table 1.

**Table 4:** Transition rates calculated from hole theory

Polymer	Ref.	*T_g_K*	${\tilde{T}}_{g}\times 10^{5}$	${\left(\partial \tilde{T}/\partial \tilde{P}\right)}_{h}\times 10^{5}$	A	B	*dT*†*_g_/dP* K/kbar
					(*∂T/∂P*)*_h_* K/kbar	(*∂T/∂P*)*_h_* K/kbar	
							
PDMSi	36, 37	150^a^	1900	2014	31.4	16.8	–
PIB	37, 38	202^b^	1800	1878	28.8	29.2	–
NR	39	204	2445	2868	–	27.6	24^c^
PP	40	244	2721	3371	35.8	38.6	–
SBR	41	256	2622	3185		36.4	–
PMA	42	281	3054	4042	–	33.8	–
PnBMA	43	293	2934	3792	44.8	40.3	24.3
PVAc	1	304	3226	4416	44.3	39.2	42.4
t-PMMA	44	320	2865	3653	40.4	42.2	35.2
PEMA	45	338	2928	3780	–	50.2	–
PET	46	340	3128	4201	–	42.2	–
PVC	47	349	3083	4104	44.9	42.3	41
PVC	48	349	3071	4079	47.4	42.1	46
PS	15	362	2811	3546	68.9	62.3	71.0
PS	47	362	2850	3623	60.2	62.1	56
PS	49	365	3138	4223	62.3	60.8	55.5
PS	50	365	2879	3681	62.6	62.7	74.2
α-PMMA	48	376	3272	4520	57.8	55.2	71
α-PMMA	47	378	3179	4312	55.1	56.8	35.9
α-PMMA	43	378	3171	4295	56.0	57.0	54.5
PCHMA	43	380	3366	4735	63.8	60.8	59.8
PoMS	50	404	3171	4295	73.4	75.1	73.0
Pcarb	51	423	3487	5020	–	66.5	–
P*α*MS^d^	56	440	3465	4967	–	81.0	52
P*α*MS (1)	52	448	3528	5118	–	83.4	–
P*α*MS (2)	52	455	3557	5189	–	86.5	–

a *T_g_* taken from ref. [58].

b *T_g_* taken from ref. [59].

c Δ*κ* determined from dynamic compressibility data [60] on vulcanized natural rubber with 12 percent combined sulfur. See text.

d Scaling factors taken the same as for P*α*MS (1).

**Table 5:** Densification rate calculations using PVT data and hole theory

Polymer	Ref.	△α × 10^4^ K^−1^	*dT_g_*/*dP* K/kbar	(*∂T*/*∂P*)*_h_* K/kbar	*κ*′ Mbar^−1^
					
PP	40	4.41	20	35.8	7.0
PnBMA	43	1.69	20.4	44.8	4.1
PVAc	1	4.32	21.2	44.3	10
*i*-PMMA	44	3.49	21.1	40.4	6.7
PVC	47	2.93	13.5	44.9	9.2
PVC	48	3.71	14	47.4	12
PS	15	2.84	31	68.9	11
PS	47	3.05	30.0	60.2	9.2
PS	49	3.15	25.0	62.3	12
PS	50	2.97	31.6	62.6	12
α-PMMA	48	3.1	18	57.8	12
α-PMMA	47	2.95	23	55.1	9.5
α-PMMA	43	2.35	23.6	56	7.6
PCHMA	43	3.38	22.4	63.8	14
PoMS	50	2.71	34.2	73.4	11
PaMS^a^	56	3.98	39	81	17

a Scaling factors taken for P*α*MS (1), Ref. [52].

**Table 6:** Densification rate calculations from dynamic data at elevated pressures and hole theory

Polymer	Ref.	△α × 10^4^ K^−1^	(*∂T*/*∂P*)*_h_* K/kbar	Ref.	Method	(*∂T*/*∂P*)*ω* K/kbar	*κ*′ Mbar^−^1
							
PIB	37, 38, 59	4.2	28.8	61	U	25	1.6
NR	39	5.40	27.6	60	C	24	1.9
PMA	42	3.7	33.8	62	D	18	5.8
PnBMA	43	1.69	40.3	63	D	16.7	4.0
PVAc	1	4.32	39.2	13	D	22	7.4
PVAc^a^	1	4.32	39.2	64	C	20	8.3
PEMA	45	2.95	50.2	65	D	20	8.9
PVC	47	2.93	44.9	66	D	18	7.9
PVC	47	2.93	44.9	67	S	16.5	8.3
PS	50	2.97	62.6	68	D	32	9.1
*α*-PMMA	43	2.35	56.0	67	S	24.5	7.4
Pcarb	51	2.81	66.5	13	D	44	6.3

a All quantities derived for same sample.

U Ultrasonic

D Dielectric

C Dynamic Compressibility

S Dynamic Shear

**Table 7:** Densification rate calculations from thermal expansion and heat capacity data and hole theory

Polymer	Kef.	*T_g_K*	V*_g_*cm^3^/g	△α × 10^4^ K^−1^	(*∂T*/*∂P*)*_h_* K/kbar	Ref.	△*C_p_*J/(g-K)	*dT_g_*/*dP*^a^ K/kbar	*κ*′ Mbar^−1^
									
PDMSi^b^	58, 30	150	0.904	6.0	31.4	27	0.30	27.0	2.6
PIB	59	202	1.072	4.2	28.8	69	.377	24.1	2.0
NR	39	204	1.023	5.40	27.6	70	.455	24.8	1.5
pp^c^	40	244	1.127	4.41	35.8	71	.51	23.8	5.3
SBR^d^	41	256	0.987	3.92	36.4	72	.456	21.7	5.8
PVAc^e^	1	304	.843	4.32	44.3	16e	.50	22.1	9.6
PVC	47	349	.729	2.93	44.9	29f	.34	21.9	6.7
PS	50	365	.976	2.97	62.6	29f	.368	28.8	10
*α*-PMMA	43	378	.864	2.35	56.0	29f	.33	23.3	7.7
Pcarb	51	423	.854	2.81	66.5	73	.22	46.1	5.7
P*α*MS^c^	56	440	.958	3.98	81.0	56	.32	52.4	11

a ^a^dT_g_/dP = T_g_V △α/△Cp.

b Partially crystalline sample.

c All quantities derived from the same sample.

d 55 percent Styrene for T_g_, V_g_ and △α, 43 percent △C_p_.

e Measurements by J. J. Weeks reported in Ref. 16.

f Average over different sources.

**Table 8:** Comparison of experimental transition rates^a^

Polymer	PVT			Dynamic		Thermal	
	Ref.	*dT_g_*/*dP*	△*κ*/△*α*	Ref.	(∂*T*/∂*P*)_ω_	Ref.	*TV*△*α*/△*C_p_*
							
PIB				61	25	59, 69	24.1
NR	39, 60		26^b^	60	24	39, 70	24.8
PP	40	20				40, 71	23.8
PVAc^c^	1	21.2	48.2	64	22	1, 16d	22.1
PVC	47	13.5	33.5	66	18	47, 29	21.9
PVC	48	14	46	67	16.5	48, 29	28.6
PS	50	31.6	74.2	68	32	50, 29	28.8
PS^c^	9	31				9	30.5
PS	55	32	73			55	34
*a*-PMMA	43	23.6	54.5	67	24.5	43, 29	23.3
*a*-PMMA^c^	9	22				9	32
Pcarb				13	44	51, 73	46.1
P*α*MS	56	39	52			56	52

a All units in K/kbar.

b △*κ* = 1.2 × 10^−5^ bar^−1^ determined from dynamic compressibility.

c All given determinations on same sample.

d Measurements by J. J. Weeks reported in Ref. [16].

**Table 9:** Estimation of maximum densification from pyrolysis data

Polymer	Ref.	Δ*H* kJ/mol	*T* K	*k(T) %*/*min*	*T_d_K*	*T_g_K*	*dT_g_*/*dP* K/kbar	P′ _max_ kbar	*κ*′ Mbar^−1^	$-\left(\frac{\Delta V}{V}\right)\max\%$

PIB	79	218	593	0.268	558	202	25	14	1.6	2
PP	79	255	623	.069	606	244	20	18	7.0	13
PMA	80	155	558	.270	515	282	18	13	5.8	8
PVAc	81, 82	112	497	5.58	409^a^	304	21.2	5	9.3	5
PVC	83	126	508	0.43	458	349	13.5	8	6.4	5
PS	80	218	608	.163	577	363	30.0	7	8.1	6
*a*-PMMA	80	230	583	.250	552	378	23.6	7	7.3	5
P*α*MS	80	243	546	.276	519	448	39	2	4.9	1

a Stated to be unstable at temperatures above 463K in Ref. [81]. During sample preparation [1] slight discoloration was observed after heating overnight in a vacuum at 403 K.

**Table 10:** Estimation of change in refractive index from densification rate

Polymer	*T_g_°C*	n_D_(T)^a^(°C)	*dn_D_*/*dT^b^ °*C^−1^	*^n^_D_(Tg)*	*f(n_D_)*	*κ*′ Mbar^−1^	*δ*′ Mbar^−1^

PIB	−71	1.51^c^	−0.0003	1.54	0.421	1.6	0.7
NR	−69	1.52(25)	−0.00037	1.55	.428	1.9	.8
PP	−29	1.49^c^	(d)	1.51	.400	4.1	1.6
SBR	−16	1.53^c^	(d)	1.54	.421	5.8	2.4
PMA	9	1.47(20)	(d)	1.47	.373	5.8	2.2
PnBMA	20	1.48(25)	(d)	1.48	.380	0.7	0.3
PVAc	31	1.48(20)	−0.0001	1.48	.380	9.1	3.5
PEMA	64	1.48(25)	(e)	1.48	.380	8.9	3.5
PVC	76	1.55^c^	(e)	1.54	.421	7.6	3.2
PS	90	1.59^c^	−0.00013	1.58	.449	8.2	3.7
α-PMMA	105	1.49(20)	−0.00012	1.48	.380	7.4	2.8
PCHMA	107	1.51(20)	−0.00013	1.50	.394	12.6	5.0
Pcarb	143	1.58^c^	(e)	1.57	.442	5.1	2.3

a Taken from Ref. [35].

b Taken from Ref. [84].

c Temperature taken as 25°C.

d *dn_D_*/*dT* taken as −0.0003.

e *dn_D_*/*dT* taken as −0.0001.

## Footnotes

1 Figures in brackets indicate literature references at the end of this paper.

2 For conversion to SI units, 1 MPa = 10 bar.

3 Differential scanning calorimetry

4 Preliminary work of this kind on 18 polymers, including PMMA. PS, Pearl), ami PVC, lias been reported by H. Eyring and coworkers in Ref. [78]. In all of these the decomposition temperature increases monotonically with pressure. We have been unable to obtain a final report on this activity.

## 6. Appendix

As indicated earlier, eqs (5) in the text involve a linearization of pertinent quantities and proximity to the transition line *T_g_(P)* in figure 3. The experimental data found in the literature often do not satisfy these conditions. Hence we reconsider here the processes depicted in figure 3, by replacing the simplifications adopted earlier by the more general form. This will not only permit the prediction of densification effects under more extreme conditions, but also allow us to gage the quantitative validity of the linearization. Clearly, a knowledge of the equations of state is required for an explicit evaluation, but is certainly not available for the wide range of systems discussed here. However we shall be able to present typical numerical illustrations using PVAc where appropriate data exist [1, 14].

From the definitions of the coefficients *α* and *κ* it follows that:

$$V/V_{0}=\exp\left[\int_{T_{0}}^{T}\alpha dT\right];V/V_{0}=\exp\left[-\int_{P_{0}}^{P}\kappa dP\right]$$

where the subscript indicates an initial value. However, it will be accurate enough to omit second and higher powers in the expansions of the exponentials. Considering an average, temperature independent value for the liquid, 〈*α*〉 = 8 × 10^−4^ K^−1^, and a temperature interval of 100 degrees, we obtain for the integral a value of 8 × 10^−2^. Thus the quadratic term changes the total result by 0.34 percent. The values were chosen so as to magnify the effect. For the glass, the approximation will be even more adequate. With a pressure difference of 2 kbar and 〈*κ*〉 = 4 × 10^−5^ bar^−1^, the magnitude of the relative volume change is the same and identical conclusions are obtained as for the α-term.

Denoting the initial temperature in the melt as *T*_0_ and the final temperature in the glass as 77, with the initial pressure taken as zero, we obtain instead of eq (3), when the pressure dependence of the α’s and the temperature dependence of the *κ*’s are taken into account, the following expression:

$$\begin{array}{l} \left(V_{A}-V_{B}\right)/V_{0}=\int_{0}^{P\prime }\left[\kappa_{l}\left(T_{0},P\right)-\kappa_{g,c}\left(T_{f},P\right)\right]dP\\ -\int_{T_{g}{\;}^{\left(0\right)}}^{T_{g_{\left(P\prime \right)}}}\left[\alpha_{l}\left(T,0\right)-\alpha_{g,c}\left(T,P\prime \right)\right]dT+\int_{T_{g}{\;}^{\left(P\prime \right)}}^{T_{0}}\left[\alpha_{l}\left(T,P\prime \right)-\alpha_{l}\left(T,0\right)dT-\int_{T_{f}}^{T_{g_{\left(0\right)}}}\left[\alpha_{g,b}\left(T,0\right)-\alpha_{g,c}\left(T,P\prime \right)\right]\right]dT \end{array}$$ (A-1)

where the subscripts *b* and *c* pertain to the low and high pressure glasses respectively. This choice conforms with the nomenclature used in refs. [1] and [14]. In the linearized derivation, *α* and *κ* for both liquid and glass are taken to be constants. Accordingly, there is no distinction between the values of *α_g,b_* and *α_g,c_*, and, similarly, *κ_g,b_* and *κ_g,c_*

To proceed further, we make use of an explicit equation of state. It is most convenient to employ the extensively tested Tait relations for both melt and glass. To recapitulate the pertinent equation (14), [50]:

$$\kappa \left(P,T\right)=C{\left\{\left[P+B\right]\times \left[1-C\ell n\left(1+P/B\right)\right]\right\}}^{-1}$$

where

B(T)=aexp(−bT)

and

α(T,P)−α(T,0)=Pκ(T,P)dℓnB/dT=−bPκ. (A-2)

The last transforms eq (A-1) into

$$\begin{array}{l} \left(V_{A}-V_{B}\right)/V_{0}=\int_{0}^{P\prime }\left[\kappa_{l}\left(T_{0},P\right)-\kappa_{g,c}\left(T_{f},P\right)\right]dP\\ -\int_{T_{g}{\;}^{\left(0\right)}}^{T_{g_{\left(P\prime \right)}}}\left[\alpha_{l}\left(T,0\right)-\alpha_{g,c}\left(T,0\right)\right]dT-\int_{T_{f}}^{T_{g_{\left(0\right)}}}\left[\alpha_{g,b}\left(T,0\right)-\alpha_{g,c}\left(T,0\right)\right]dT-b_{l}P\prime \int_{T_{g\left(P\prime \right)}}^{T_{0}}\kappa_{l}\left(T,P\prime \right)\times dT-b_{g,c}P\prime \int_{T_{f}}^{T_{g\left(P\prime \right)}}\kappa_{g,c}\left(T,P\prime \right)dT \end{array}$$ (A-3)

where the first two terms will predominate.

We now proceed to evaluate the integrals in eq (A-3) which are identified as follows:

$$\begin{array}{l} I_{1}=\int_{0}^{P\prime }\left[\kappa_{l}\left(T_{0},P\right)-\kappa_{g,c}\left(T_{f},P\right)\right]dP\to \Delta \kappa P\prime \\ I_{2}=-\int_{T_{g}{\;}^{\left(0\right)}}^{T_{g\left(P\prime \right)}}\left[\alpha_{l}\left(T,0\right)-\alpha_{g,c}\left(T,0\right)\right]dT\to -\Delta \alpha \left(dT_{g}/dP\right)P\prime \\ I_{3}=-\int_{T_{f}}^{T_{g}{\;}^{\left(0\right)}}\left[\alpha_{g,b}\left(T,0\right)-\alpha_{g,c}\left(T,0\right)\right]dT\to 0\\ I_{4}=-b_{l}P\prime \int_{T_{g}}^{T_{0^{\left(P\prime \right)}}}\kappa_{l}\left(T,P\prime \right)dP\to 0\\ I_{5}=-b_{g,c}P\prime \int_{T_{f}}^{T_{g^{\left(P\prime \right)}}}\kappa_{g,c}\left(T,P\prime \right)dT\to 0 \end{array}$$ (A-4)

where the terms to the right of the arrows are the corresponding linear approximations used in eqs (5) in the text.

Since *C* = 0.0894, the compressibility may be written in good approximation, and consistent with the expansion of the exponentials above, as *C/(B* + *P*). Thus we find for *I*_1_,

$$I_{1}=C\ell n\left(\frac{1+P\prime /B_{l}\left(T_{0}\right)}{1+P\prime /B_{g,c}\left(T_{f}\right)}\right).$$

*I_2_* and *I_3_* are evaluated by expressing the atmospheric pressure volume as *V*_0_ *= A*_0_ + *B*_0_*T + C*_0_*T^2^* for which *α* = (*B*_0_ + 2C_0_T)/V_av_ is a good approximation. *V*_av_ is taken as the average of the two bounds. Finally the general integral corresponding to *I*_4_ and *I*_5_ is

∫κ(T,P)dT=(C/P){T+(1/b)ℓn[P+aexp(−bT)]}+f(P).

Using the parameters for PVAc given in tables 1 and 2 of ref. [14], the values of the integrals and their linearized counterparts are summarized in table A-1. From ref. [1] *T_g_*(0) = 30.7 °C and *T_g_*(*P*′) = 48.0 °C. *T*_0_ and *T_f_ are* taken to be 90 and 0 C. These two temperatures are considered to be sufficiently remote from *T_g_*(*P*), to be characteristic of the equilibrium and glassy states, respectively. The total relative volume differences are given at the bottom of the table followed by the corresponding densification rates. The difference between the two values of κ′ amounts to about 4 percent, which is quite satisfactory, since the experimental error on this quantity appears to be considerably larger. This good agreement seen in table A-1 arises however from a cancellation of approximation errors. Moreover, it is gratifying, that the value *κ′* = 8.8 Mbar^−1^, based on the Tait equation is essentially identical to that obtained by directly measuring the volume difference. (See sec. 4.3.) This illustrates the satisfactory performance of the analytical expressions in representing the experimental data.

**Table A-1:** Values of integrals in eqs (A-4)

*i*	Integral Value, *I_i_*	Linear Counterpart
		
1	2 042 × 10^−2^	1.465 × 10^−2^
2	−0.649 × 10^−2^	−0 733 × 10^−2^
3	0.106 × 10^−2^	0
4	−0.552 × 10^−2^	0
5	−0.242 × 10^−2^	0
		
(*V_A_*−*V_b_*)*/v_o_*	0.705 × 10^−2^	0.732 × 10^−2^
*κ*′ (Mbar^−1^)	8.8	9.2
